# *Helicobacter pylori* Eradication Treatments and Risk of Alzheimer Disease: A Case–Control Study Nested in the Finnish Population

**DOI:** 10.1097/EDE.0000000000001831

**Published:** 2025-01-27

**Authors:** Emmi Keränen, Jaana Rysä, Miia Tiihonen, Sirpa Hartikainen, Anna-Maija Tolppanen

**Affiliations:** From the ^a^School of Pharmacy, University of Eastern Finland, Kuopio, Finland.

**Keywords:** Alzheimer disease, Case–control study, Confounding factors, Epidemiologic, *Helicobacter pylori*, Risk factors

## Abstract

**Background:**

*Helicobacter pylori* (*H. pylori*) has been inconsistently associated with the risk of Alzheimer disease. The exposure assessment period has often overlapped with the prodromal time of Alzheimer disease. Cognitive disorders might increase vulnerability to infectious pathogens, complicating the ascertainment of the temporal relationship between *H. pylori* infection and Alzheimer disease.

**Methods:**

This Finnish nested case–control study included 70,520 persons with incident Alzheimer disease diagnosed between 2005 and 2011 and 281,233 age-, sex-, and region of residence-matched controls. We obtained information on comorbidities and drug use from the national healthcare registers. We identified dispensed *H. pylori* eradication treatments from the Prescription Register. We considered exposure at least 5 years before Alzheimer disease diagnosis in the main analysis. We compared the risk of Alzheimer disease between *H. pylori* eradication treatment users and nonusers using confounder-adjusted (comorbidities and other drug use) conditional logistic regression. We assessed cumulative exposure by calculating the number of eradication treatments.

**Results:**

The prevalence of exposure to *H. pylori* eradication treatment at least 5 years before the outcome was 4.1% in cases and 3.9% in controls. The odds ratio (95% confidence interval) was 1.06 (1.02, 1.11) in the crude and 1.03 (0.99, 1.07) in the confounder-adjusted model. We observed no association between cumulative exposure and risk of Alzheimer disease.

**Conclusion:**

Our results, reflecting diagnosed and treated *H. pylori* infection late in life, do not support the hypothesis of *H. pylori* as an independent risk factor for Alzheimer disease. The previously reported association may be explained by reverse association and confounding.

The number of persons with dementia is growing quickly, especially in countries with demographic transition due to population aging,^1^ with Alzheimer disease (AD) being the most common cause.^2^ The current treatment strategies for AD merely ameliorate the symptoms. Therefore, it is important to identify novel risk factors and effective preventive measures. Previous studies have proposed a link between pathogenic micro-organisms^3^ including *Helicobacter pylori*, a Gram-negative bacterium that inhabits the human stomach,^4^ and risk of AD.

Affecting approximately half of the world’s adult population and prevalent especially in developing countries,^5^ *H. pylori* is a well-known risk factor for gastroduodenal pathologies and it has been proposed to directly or indirectly contribute to an increased risk of extragastric and neurodegenerative disorders.^6^ Two small prospective French cohort studies reported an association between *H. pylori* exposure and higher risk of dementia,^7,8^ and recently, a nested case–control study based on UK electronic health records observed a higher risk of AD among those with clinically apparent *H. pylori* infection.^9^ A case–control study reported an association between *H. pylori* seropositivity and AD^10^; another reported higher cerebrospinal fluid concentrations of *H. pylori* antigens in persons with AD in comparison to age-matched controls with normal cognition.^11^ However, the long prodromal period of AD and the unclarity of exposure assessment time in previous studies complicate the interpretation of those findings.

A small prospective study suggested that a triple eradication antibiotic regimen to eliminate *H. pylori* was associated with better cognitive and functional status in persons with AD,^12^ and successful eradication treatment was associated with better survival during a 5-year follow-up period in another cohort of persons with AD.^13^ However, these findings may reflect the overall beneficial impact of treating comorbidities in AD. There are no other studies assessing *H. pylori* eradication treatments in terms of cognitive disorders, but the eradication treatments typically consist of two concomitant oral antibiotics and a proton pump inhibitor (PPI). The findings of studies assessing antibiotics or PPIs as individual risk factors for dementia or AD have been inconsistent.^14–16^ We investigated the association between *H. pylori* eradication treatment and the risk of AD in a nested case–control study of Finnish community-dwelling persons.

## METHODS

### Study Design and Study Population

The Medication Use and Alzheimer Disease (MEDALZ) study is a nationwide nested case–control study including 70,718 Finnish community-dwelling individuals diagnosed with AD during 2005–2011 (AD cases) and up to four matched comparison individuals by age, sex, and region of residence (n = 282,845). Controls were matched on the AD diagnosis date (index date). The study and data sources have been described in detail previously.^17^

We excluded 197 cases and 788 controls with a diagnosis of stomach cancer (diagnosis before the index date) in this study. A detailed definition of exclusion criteria is provided in eTable 1; http://links.lww.com/EDE/C213. In addition, we excluded 824 controls and one case, as they did not have the matched cases or controls after the stomach cancer exclusions. The final study population included 70,520 AD cases and 281,233 matched controls (eFigure 1; http://links.lww.com/EDE/C213).

The maintainers of the applied registers used personal identification numbers to link information across registers and pseudonymized all data before submission to the research team. According to the Finnish legislation, approval of the ethics committee or informed consent was not required. The MEDALZ study plan was approved by the register maintainers and the approval was updated by the Finnish Social and Health Data Permit Authority Findata.

### Alzheimer Disease Diagnosis

Information on AD diagnosis was obtained from the special reimbursement register. AD diagnoses were confirmed by a geriatrician or neurologist and based on the National Institute of Neurological and Stroke and the Alzheimer Disease and Related Disorders Association and Diagnostic and Statistical Manual of Mental Disorders, 4th Edition criteria.^18,19^ A clinical professional evaluated diagnostic evidence, consisting of anamnestic information from the patient and their caregivers, findings from clinical examination (including CT and MRI scan and the exclusion of alternative diagnoses), and all diagnostic findings to meet the prespecified criteria. The Finnish Current Care guidelines for cognitive disorders recommend that antidementia drugs be initiated in all patients diagnosed with AD in a mild or moderate state if there is no contraindication for use.^20^ Social Insurance Institution requires a clinically verified diagnosis of a mild or moderate state of AD to grant special reimbursement for antidementia drug purchases.

### Exposure to *Helicobacter pylori* Eradication Treatment

In Finland, the first-line *Helicobacter pylori* eradication regime is the triple treatment with concomitant administration of two oral antibiotics and a PPI. We identified exposure to eradication of *H. pylori* from the Finnish Prescription Register using Anatomical Therapeutic Chemical-classification system (ATC) code A02BD starting from the beginning of the register in 1995 to the index date or until the beginning of the lag period. Dispensed eradication treatments during the study period are listed in eTable 2; http://links.lww.com/EDE/C213. Cumulative exposure was assessed using a defined daily dose (DDD). DDD is the assumed average maintenance dose per day for a drug used for its main indication in adults, assigned by the World Health Organization Collaborating Centre in Oslo. For *H. pylori* eradication treatment, seven DDDs typically represent a single course of treatment.

We assessed exposure in two different time windows as we wanted to account for increased healthcare contact before AD diagnosis among cases, which could increase the likelihood of diagnosing and treating other comorbidities. We categorized the exposure based on whether it had occurred at least 5 years before the index date or within the 5-year period before the index date (lag period). We categorized exposure both for binary (exposed vs. not) and cumulative DDDs. The mean duration of exposure assessment time in cases and controls was 8.7 years until the lag time, and the mean duration of exposure assessment until the index date was 13.7 years.

### Confounders

We considered comorbidities (cardiovascular disease, stroke, diabetes, stomach ulcer, asthma/chronic obstructive pulmonary disease, cancer, impatient care with psychiatric diagnoses, and substance abuse) and reimbursed prescription of vitamin B12 and benzodiazepine and a related drug, antidepressant, antipsychotic, and PPI use as confounders. Information on them was obtained from the beginning of the data source until 5 years before the index date. Details on the data source, time periods, and coding systems applied are provided in eTable 1; http://links.lww.com/EDE/C213. We then categorized the number of different ATC codes in 1995 into four groups: none, 1–2, 3–5, and 6 or more. We obtained information on the highest occupational social class from Statistics Finland and classified it as managerial/professional, office, farming/forestry, sales/industrial/cleaning, and unknown.

We calculated the following proxies of healthcare contacts during the 5-year lag period for descriptive analyses on whether the exposed persons had more healthcare contacts during the lag period: sum of purchased drugs (0–1, 2–5, 6–9, 10–14, 15–19, and 20 or more), hospital days (0, 1–7, 8–30, and 31 or more), and polyclinic visits in specialized healthcare (1–2, 3–5, 6–10, 11–25, and 25 or more).

### Statistical Analyses

We evaluated differences between cases and controls as well as users and nonusers by the two-sample *T* test or linear regression (continuous variables with normal distribution), Mann–Whitney *U* test or Kruskal–Wallis test (continuous variables with skewed distribution), and Pearson chi-squared test (categorical variables). We used conditional logistic regression adjusted for confounders and mediators to evaluate the association between *H. pylori* eradication treatment and AD. We investigated the risk of AD between users and nonusers of *H. pylori* eradication treatment, considering the timing of the exposure (lag only, before lag only, or both). We conducted sensitivity analysis for user–nonuser comparison, varying the lag time between 1-year intervals (0–9 year lag) with nonoverlapping assessment of confounders and exposure to *H. pylori* eradication treatment in 1996–2011.

Furthermore, we conducted dose–response analyses for the risk of AD, with exposure categorized to 7, 14, or 21 or more DDDs, both before 5-year lag period and before and at 5-year lag period. Stata MP14.0 (StataCorp, College Station, TX) was used to perform all analyses.

## RESULTS

### Characteristics of the Study Population

The mean age of cases and controls was 80 years and 65% were women (Table 1). Comorbidities and the number of different prescription drugs in use were more common among persons with AD than those without AD. The cases also had a higher sum of purchased prescription drugs, hospital days, and polyclinic visits to specialized healthcare during the 5-year lag period, indicating more healthcare contacts in this time window.

**TABLE 1.** Characteristics of Cases With Alzheimer Disease and Controls Without Alzheimer Disease

	Cases n = 70,520	Controls n = 281,233
Age at index date, mean (95% CI)	80.1 (80.0, 80.1)	80.0 (80.0, 80.0)
Women, n (%)	45,997 (65.2)	183,526 (65.3)
Highest occupational social class, n (%)		
Managerial/professional	14,662 (20.8)	80,235 (21.4)
Office	5,995 (8.4)	23,438 (8.3)
Farming, forestry	13,393 (19.9)	55,135 (19.6)
Sales, industrial, cleaning	30,064 (42.6)	110,189 (39.2)
Unknown	6,446 (9.1)	32,236 (11.5)
Cardiovascular disease, n (%)	24,400 (34.6)	91,462 (32.5)
Stroke, n (%)	1,565 (2.2)	5,843 (2.1)
Diabetes, n (%)	3,583 (5.1)	10,333 (3.7)
Stomach ulcer, n (%)	1,361 (1.9)	4,591 (1.6)
Asthma/chronic obstructive pulmonary disease, n (%)	4,026 (5.7)	15,443 (5.5)
Cancer, n (%)	5,012 (7.1)	19,081 (6.8)
Inpatient care with psychiatric diagnoses, n (%)	3,467 (4.9)	12,596 (4.5)
Substance abuse, n (%)	1,015 (1.4)	3,564 (1.3)
Benzodiazepine and related drug use^a^, n (%)	11,683 (16.6)	41,837 (14.9)
Antidepressant use^a^, n (%)	3,454 (4.9)	11,125 (4.0)
Antipsychotic use^a^, n (%)	1,501 (2.1)	5,430 (1.9)
Use of proton pump inhibitors^a^, n (%)	1,741 (2.5)	6,422 (2.3)
Reimbursed prescription of vitamin B12^a^, n (%)	451 (0.64)	1,568 (0.56)
Categorized drug sum^a^^,^^b^, n (%)		
None	16,419 (23.3)	76,400 (27.2)
1–2	18,145 (25.7)	71,813 (25.5)
3–5	19,616 (27.8)	75,597 (26.9)
6 or more	16,340 (23.2)	57,423 (20.4)
Proxies for healthcare contact in lag time		
Sum of purchased drugs, n (%)		
0–1	2,647 (3.8)	20,314 (7.2)
2–5	10,235 (14.5)	43,492 (15.5)
6–9	14,889 (21.1)	59,239 (21.1)
10–14	17,979 (25.5)	68 057 (24.2)
15–19	12,285 (17.4)	45,433 (16.2)
20 or more	12,485 (17.7)	44,698 (15.9)
Hospital days, n (%)		
0	16,468 (23.4)	94,100 (33.5)
1–7	17,871 (25.3)	82,805 (29.4)
8–30	18,600 (26.4)	64,412 (22.9)
31 or more	17,581 (24.9)	39,916 (14.2)
Polyclinic visits to specialized healthcare, n (%)		
None	8,070 (11.4)	57,476 (20.4)
1–2	12,772 (18.1)	50,520 (18.0)
3–5	15,313 (21.7)	55,617 (19.8)
6–10	15,566 (22.1)	53,219 (18.9)
11–25	14,541 (20.6)	48,635 (17.3)
25 or more	4,258 (6.0)	15,766 (5.6)
Categorized exposure, n (%)		
None	65,434 (92.8)	261,872 (93.1)
Lag only	2,132 (3.0)	8,184 (2.9)
Before lag only	2,879 (4.1)	10,851 (3.9)
Both	75 (0.1)	326 (0.1)
Defined daily doses (DDDs) before 5-year lag, n (%)		
7	2,734 (92.6)	10,325 (92.4)
14	194 (6.6)	745 (6.7)
21 or more	26 (0.9)	107 (1.0)
DDDs in 5-year lag, n (%)		
7	2,077 (94.1)	8,010 (94.1)
14	115 (5.2)	445 (5.2)
21 or more	15 (0.7)	55 (0.7)
DDDs in total, n (%)		
7	4,687 (92.2)	17,802 (92.0)
14	344 (6.8)	1,319 (6.8)
21 or more	55 (1.1)	240 (1.2)

a In 1995.

b Excluding A02BD (combinations for eradication of *H. pylori*).

Altogether 7.2% of cases and 6.9% of controls had at least one eradication treatment purchase before the index date, with 4.1% of cases and 3.9% of controls having the first exposure before the 5-year lag. Over 90% of the exposed had just one course of treatment (cumulative exposure seven DDDs).

The characteristics of *H. pylori* eradication users and nonusers are shown in eTable 3; http://links.lww.com/EDE/C213. There was no difference in age at the index date. Those exposed to *H. pylori* eradication treatment had a higher prevalence of other drug use and a higher number of prescription drug purchases in the first year of exposure assessment. The only exception was purchased vitamin B12 prescriptions, which were more frequent in nonusers although the difference was small. Likewise, there was a higher prevalence of comorbidities in users, except for stroke, which was more frequently observed in nonusers. Overall healthcare contacts during lag time were more frequent in users. Nonusers had purchased fewer prescription drugs and had less hospital days and polyclinic visits to specialized healthcare during lag time.

Characteristics of *H. pylori* eradication treatment users categorized by the time of use are shown in eTable 4; http://links.lww.com/EDE/C213. Prevalence of prescription drug use, comorbidities, and number of healthcare contacts were similarly distributed between users and nonusers. Those who had used *H. pylori* eradication treatments both before and during the lag time were younger than other participants, but they had the highest number of prescription drug purchases in the first year of exposure assessment.

### Association Between *Helicobacter pylori* Eradication Treatment and Alzheimer Disease

The odds ratio (OR) (95% confidence interval [CI]) for *H. pylori* exposure in the main exposure period only was 1.06 (1.02, 1.11) in the crude model that accounted for factors included in matching (Table 2). Additional adjustment for confounders had a minor impact on point estimate (OR: 1.03, 95% CI: 0.99, 1.07). Use during lag time only was not associated with AD, neither was the use before and during the lag, although the number of persons exposed in both time windows was small (0.11% of cases and 0.12% of controls).

**TABLE 2.** Analysis of Association Between *H. pylori* Eradication Treatment and Alzheimer Disease

	Cases Total n = 70,520 n (%)	Controls Total n = 281,233 n (%)	Crude OR (95% CI)^a^	Adjusted OR (95% CI)^b^
No use	65,434 (92.8)	261,872 (93.1)	1.00 (reference)	1.00 (reference)
Lag only	2,132 (3.0)	8,184 (2.9)	1.04 (0.99, 1.09)	1.02 (0.97, 1.07)
Before lag only	2,879 (4.1)	10,851 (3.9)	1.06 (1.02, 1.11)	1.03 (0.99, 1.07)
Both	75 (0.11)	326 (0.12)	0.92 (0.72, 1.18)	0.88 (0.69, 1.13)

a Conditional logistic regression accounting for matching characteristic age, sex, and university hospital district.

b Crude model with additional adjustment for highest occupational class, cardiovascular disease, stroke, diabetes, stomach ulcer, asthma, cancer, inpatient care with psychiatric diagnoses, substance abuse, use of benzodiazepines and related drugs, antidepressants, antipsychotics, proton pump inhibitors, reimbursed vitamin B12 prescriptions, and categorized sum of purchased drugs in 1995.

The findings from sensitivity analyses using 0–9 year lag times and nonoverlapping confounders and exposures were in line with the main results (eFigure 2; http://links.lww.com/EDE/C213).

We observed no association between cumulative exposure, defined as a number of eradication treatments, and the risk of AD, regardless of the period for cumulative exposure calculation (Table 3).

**TABLE 3.** Association Between Alzheimer Disease and *H. pylori* Eradication Treatment DDDs Before and Both Before and at 5-Year Lag Time, Compared With Seven DDDs

Before Lag Time	Cases Total n = 2,954 n (%)	Controls Total n = 11,177 n (%)	Crude OR (95% CI)^a^	Adjusted OR (95% CI)^b^
7 14 21 or more	2,734 (92.6) 194 (6.6) 26 (0.88)	10,325 (92.4) 745 (6.7) 107 (0.96)	1.00 (reference) 1.00 (0.85, 1.18) 0.94 (0.61, 1.44)	1.00 (reference) 0.99 (0.84, 1.17) 0.94 (0.61, 1.44)
**Before and Within 5-Year Lag**	**Cases** **Total n = 5,086** **n (%)**	**Controls** **Total n = 19,361** **n (%)**	**Crude OR (95% CI)^a^**	**Adjusted OR (95% CI)^b^**
7 14 21 or more	4,687 (92.2) 344 (6.8) 55 (1.1)	17,802 (92.0) 1,319 (6.8) 240 (1.2)	1.00 (reference) 0.99 (0.88, 1.12) 0.87 (0.64, 1.16)	1.00 (reference) 0.99 (0.87, 1.12) 0.87 (0.65, 1.17)

a Conditional logistic regression accounting for matching characteristic age, sex, and university hospital district.

b Crude model with additional adjustment for highest occupational class, cardiovascular disease, stroke, diabetes, stomach ulcer, asthma, cancer, inpatient care with psychiatric diagnoses, substance abuse, use of benzodiazepines and related drugs, antidepressants, antipsychotics, proton pump inhibitors, reimbursed vitamin B12 prescriptions, and categorized sum of purchased drugs in 1995.

## DISCUSSION

We observed no association between *H. pylori* eradication treatments and AD in our nested nationwide case–control study, contrary to previously reported higher risk of dementia/AD in persons with serologically confirmed *H. pylori* infection,^7,8,21^ infection confirmed both by serology and histopathological examination,^10^ or clinically apparent infection.^9^

*H. pylori* was associated with an increased risk of all-cause dementia in a meta-analysis of 10 studies by Liu et al.^22^ The pooled point relative risk estimates were suggestive of increased risk of AD in cohort studies (1.33, 95% CI: 0.86, 2.05) and case–control studies (1.72, 95% CI: 0.97, 3.04). There was substantial between-study heterogeneity (e.g., ethnicity, *H. pylori* detection methods), and two studies with positive findings were not adjusted for confounders. Alternatively, the lack of association with AD in the meta-analysis may be due to a lack of statistical power. Study samples were between 61 and 83,965 participants and the total number of persons in the meta-analyses was 95,459 for cohort studies and 1,102 for case–control studies. In another meta-analysis by Fu et al.,^23^ *H. pylori* infection was associated with an increased risk of dementia (1.40, 95% CI: 1.12, 1.76), but in the subgroup analyses the association was seen only in the studies conducted in Asia (1.60, 1.20, 2.15) but not in Europe (1.09, 0.91, 1.31).

A recent nested case–control study based on UK Clinical Practice Research database including 40,455 AD cases observed a higher risk of AD among those with *H. pylori* exposure, which was assigned based on a positive *H. pylori* test, eradication treatment, and *H. pylori*-related disease or related complication.^9^ In that study, despite the OR indicating a higher risk of AD among the exposed (adjusted OR: 1.11, 95% CI: 1.01, 1.21), the difference in exposure prevalence between cases and controls was relatively small (1.3% and 1.1%, respectively). Those results were adjusted for specific comorbidities and medications, which may not fully capture the overall comorbidity burden and residual confounding may have remained. We used the sum of prescription drugs in the beginning of the exposure assessment as a proxy of comorbidity and contact with prescribers. The mean follow-up or exposure assessment periods in that study and our study were similar (Douros et al. 11 years, our study 13 years).

Although our exposure definition, *H. pylori* eradication treatment administered in older age, does not reflect a lifetime history of *H. pylori* exposure, our findings are in line with previous studies that ascertained *H. pylori* exposure based on seropositivity^24,25^ or diagnosed *H. pylori* infection.^26^ Neither a Dutch cohort study^24^ nor a Chinese case–control study^25^ observed an association between *H. pylori* infection and increased risk of AD. Moreover, a Taiwanese cohort study observed no association with AD; however, *H. pylori* exposure was associated with a higher risk of non-AD dementia.^26^ These studies were included in the meta-analysis by Liu et al.^22^

*H. pylori* serology is considered a well-suited exposure definition for epidemiologic studies, although in populations with a low prevalence of *H. pylori* infection, serology can cause false-positive results due to its low specificity and as a method, it cannot differentiate between acute and past infections.^27,28^ As some of the studies performed serologic testing in persons already diagnosed with AD or dementia,^10,11^ it is possible that the *H. pylori* infections were recent and thus their timing was not appropriate for assessing a causal relationship. Only one study has utilized a lag period: the recent nested case–control study by Douros et al.^9^ considered *H. pylori* exposure that had been recorded at least 2 years before the index date. In that study, AD diagnosis was based on the recorded diagnostic code for AD and prescribed AD medications. In case of multiple events qualifying the person to AD diagnosis, the date of AD diagnosis corresponded to the date of the last event,^9^ meaning that the applied lag time may not have been sufficient to control for possible detection bias.

We accounted for the prodromal period of AD^29^ by using a lag of 5 years, based on our earlier observations on increased healthcare contacts among people who were later diagnosed with AD in this time window.^30^ The use of lag time should also avoid the possible concern of differential underdiagnosis between persons with and without AD due to cognitive alterations. If diagnosis and consequent *H. pylori* eradication were less common in AD cases already during the exposure assessment, that would lead to an underestimation. However, all our AD cases had to be in the mild or moderate stage at the date of AD diagnosis, and due to the application of 5-year lag, it is unlikely that they were unable to express their discomfort. On the other hand, healthcare contact due to *H. pylori* eradication prescription may increase the likelihood of being diagnosed with other conditions, including AD, and our design also accounts for this detection bias.

As the association between *H. pylori* eradication and AD was not observed after adjusting for comorbidities associated with a higher risk of AD,^31^ other drug use, such as antidepressants or benzodiazepines also associated with increased AD risk,^32,33^ and sum of prescription drugs as a proxy for healthcare contact, the crude association in our study seems to be explained by the higher prevalence of these factors among the *H. pylori* exposed.

Most of the previous epidemiologic studies on the association between *H. pylori* infection and the risk of AD or dementia, including those reporting a positive association,^7,8,10,21^ have been relatively small, which may result in selected populations contrary to our nationwide study. However, the generalizability of our results to other geographical locations with distinct prevalence and virulence of the *H. pylori* bacterium to ours may be limited. Interestingly, our results regarding AD were similar to the aforementioned Dutch study, where there was a relatively low prevalence of *H. pylori* and less virulent strains.^23^ Like ours, a Chinese study conducted in Chongqing province^24^ showed no association between *H. pylori* and AD, although the total infectious burden containing other pathogens alongside *H. pylori* was associated with a higher risk of AD. However, both in mainland China and locally in Chongqing province the prevalence of *H. pylori* infection is higher^34^ and there might be more virulent strains in China in comparison to European countries, as approximately 60% of *H. pylori* is CagA-positive in Western countries whereas the prevalence is >90% in Asia.^35^

The biologic mechanism connecting *H. pylori* and AD is widely unknown, although several neuroinflammation-related mechanisms have been hypothesized.^36–38^ Evidence from animal experiments is inconsistent: although an intraperitoneal injection of *H. pylori* filtrate appeared to induce memory and spatial learning deficits in rats,^39^ long-term helicobacter infection did not induce amyloid plaques or neuroinflammation in mice.^40^ Previous evidence suggests that *H. pylori* may contribute to AD pathogenesis as a part of a larger entity of accumulated infections, measured as the infectious burden.^24^ Alongside modulating the gastric microenvironment, *Helicobacter pylori* infection can also influence host-pathogen interactions with other pathogens.^4^ On the other hand, individuals with major cognitive disorders might be more vulnerable to infectious pathogens, complicating the ascertainment of the temporal relationship between *H. pylori* and AD.

Strengths of our study included the use of nationwide comprehensive registers, with appropriate data validity for epidemiologic analyses and long-term exposure assessment. We took into consideration bias caused by reverse causality and detection. We assessed the role of cumulative exposure. The use of international criteria and the requirement of a clinically verified diagnosis of AD ensured the reliability of the outcome definition. We based drug exposure on dispensed drugs, which was more likely to reflect true exposure than prescriptions. Limitations of our study include a lack of data on primary care visits, ethnicity, and lifestyle-related confounders that would have been needed for better confounder adjustment and some residual confounding may remain. Thus, the true association in our study may be even closer to one than the reported ORs. We used occupational social class and comorbidities as surrogates for lifestyle-related confounders. Ethnicity affects both the risk of developing AD and acquiring *H. pylori* infection^36,41^ but in this age group of the MEDALZ cohort, most of our participants were of Caucasian origin. Adjustment for socioeconomic positions may also partly account for the differences observed in the risk of these conditions in different ethnic groups. The availability and reimbursement of vitamin B12 prescriptions in our data sources have changed throughout this study period and the coverage may be underestimated. However, the bias is likely to be small and nondifferential. An important limitation of our study is that the information on eradication treatments was available from 1995 onwards. Therefore, it is possible that some persons with *H. pylori* infection might have been treated before that, leaving some *H. pylori* infections unidentified in our cohort. The infection is presumably acquired during childhood or adolescence^42^ and most *H. pylori* carriers remain asymptomatic for years or decades although the infection habitually causes chronic gastritis,^43^ yet an asymptomatic infection can also affect the systemic processes of the body.^4^ Still, our findings are informative on the association between late-life *H. pylori* eradication treatment and AD.

In conclusion, our results on *H. pylori* infection diagnosed and treated in late life do not support earlier findings suggesting that *H. pylori* might be an independent risk factor for AD.
